# Reactive Oxygen Species: A Key Hallmark of Cardiovascular Disease

**DOI:** 10.1155/2016/9152732

**Published:** 2016-09-28

**Authors:** Nisha Panth, Keshav Raj Paudel, Kalpana Parajuli

**Affiliations:** Department of Pharmacy, School of Health and Allied Sciences, Pokhara University, Dhungepatan, Kaski 33701, Nepal

## Abstract

Cardiovascular diseases (CVDs) have been the prime cause of mortality worldwide for decades. However, the underlying mechanism of their pathogenesis is not fully clear yet. It has been already established that reactive oxygen species (ROS) play a vital role in the progression of CVDs. ROS are chemically unstable reactive free radicals containing oxygen, normally produced by xanthine oxidase, nicotinamide adenine dinucleotide phosphate oxidase, lipoxygenases, or mitochondria or due to the uncoupling of nitric oxide synthase in vascular cells. When the equilibrium between production of free radicals and antioxidant capacity of human physiology gets altered due to several pathophysiological conditions, oxidative stress is induced, which in turn leads to tissue injury. This review focuses on pathways behind the production of ROS, its involvement in various intracellular signaling cascades leading to several cardiovascular disorders (endothelial dysfunction, ischemia-reperfusion, and atherosclerosis), methods for its detection, and therapeutic strategies for treatment of CVDs targeting the sources of ROS. The information generated by this review aims to provide updated insights into the understanding of the mechanisms behind cardiovascular complications mediated by ROS.

## 1. Chemical Characteristics of Reactive Oxygen Species (ROS)

**Figure 1:**
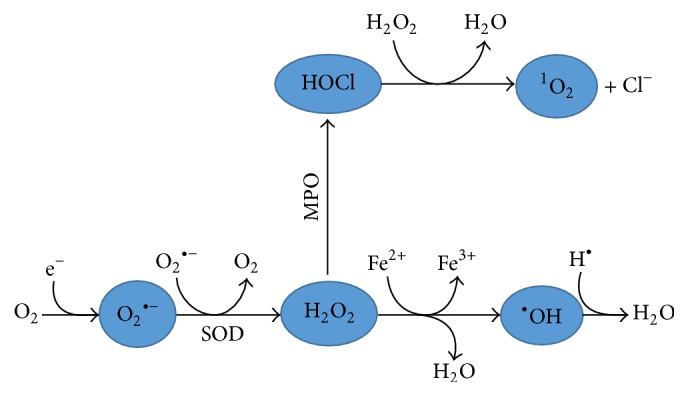
Production of ROS. The figure shows the pathway of ROS production in the human body with various enzymes involved. SOD: superoxide dismutase; MPO: myeloperoxidase.

Researchers have been continuously studying the potential role of oxidative damage in cardiovascular diseases (CVDs) for a few decades. In a simple term, the common risk factors for CVDs like diabetes mellitus, smoking, aging, hypercholesterolemia, and nitrate intolerance can further increase the possibility of the generation of ROS. Furthermore, these risk factors can trigger several pathways such as apoptosis of endothelial cells (EC), expression of adhesion molecules, activation of metalloproteinases, induction of proliferation and migration of smooth muscle cells, lipid peroxidation, and change in vasomotor functions, collectively leading to CVDs [1, 2]. ROS are chemically reactive molecules containing oxygen. Several ROS with unpaired electrons, for instance, superoxide anion (O_2_^∙−^), hydroxyl radical (OH^∙−^), and lipid radicals, are considered as free radicals. ROS, such as hydrogen peroxide (H_2_O_2_), peroxynitrite (ONOO^−^), and hypochlorous acid (HOCl), are not free radicals but possess an oxidizing effect resulting in oxidant stress. A chain reaction leads to the production of many reactive oxygen species from one ROS (Figure 1). For example, the reactions of radicals and fatty acids (polyunsaturated fatty acids, PUFAs) within the cytoplasmic membrane result in a fatty acid peroxyl radical which can attack the adjacent side chain of the fatty acid and commence production of other lipid radicals. Lipid radicals generated in this chain reaction get collected in the plasma membrane and may have an innumerable effect on cell function, including alteration in cell membrane permeability and dysfunction of membrane-bound receptors [1, 3].

## 2. Potential Sources of ROS for CVDs

**Figure 2:**
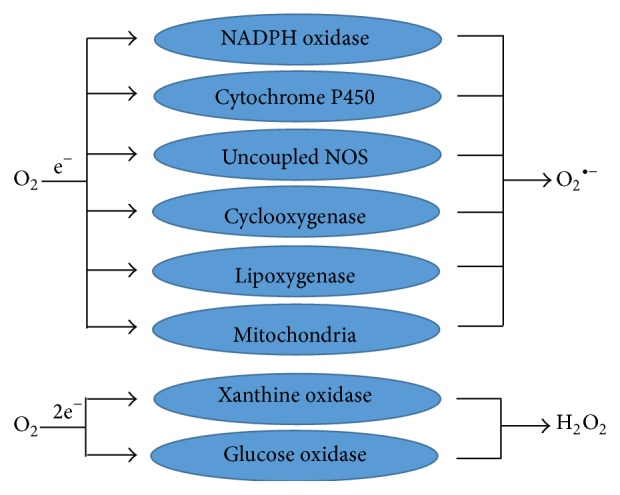
Sources of O_2_^∙−^ and H_2_O_2_ in cells. The figure shows the enzymatic pathway of superoxide anion (O_2_^∙−^) and hydrogen peroxide (H_2_O_2_) generation in cells.

In a physiological system, the imbalance between antioxidant defense mechanism and ROS production leads to oxidative stress and subsequent pathological conditions [4]. Most prominent ROS causing toxic insult to the human body are H_2_O_2_, O_2_^∙−^, ^∙^OH, and ONOO^−^ [5]. In the blood vessel wall, each layer can produce ROS in pathological conditions [6]. Wattanapitayakul and Bauer reported that, within mitochondria, oxygen is usually utilized for energy production (in the form of ATP) and oxidative phosphorylation. During the mitochondrial electron transport (MET), harmful ROS are formed but they are balanced by antioxidant defense. However, in case of ischemia or hypoxia, MET is imbalanced, leading to ATP depletion, acidosis, mitochondrial depolarization, collection of noxious metabolites, intracellular Ca^2+^ overload, and cell death [7]. For example, approximately 1–3% of molecular oxygen is converted to unstable/reactive O_2_^∙−^ in mitochondrial complexes I and III through a pathway involving oxidative phosphorylation [8]. In general, cardiac myocytes consume a high level of oxygen due to considerable higher number of mitochondria than other cells [9]. For this reason, cardiac myocytes also release ROS and cause oxidative stress to other cells [10]. But ROS do not have only a negative side, since production of ROS at physiological levels promotes cellular activities, controls the hormone level, maintains chemical balance, strengthens synaptic plasticity, and induces enzymes. Moreover, ROS also helps to fight against invading pathogens and induce an immune response against the pathogenic influence [5]. To a certain extent, ROS are neutralized by intracellular antioxidant enzymes such as glutathione peroxidase (GPx), superoxide dismutase (SOD), and catalase and consumption of other nonenzyme antioxidants like *β*-carotene, ascorbic acid, and tocopherols as a supplement [7]. In spite of being necessary to carry out cell signaling pathways, overproduction of ROS leads to injury of the cell membrane integrity causing altered permeability, change in proteins expression, and DNA damage [11]. For the majority of CVDs, the enzymatic sources of ROS include NAD(P)H oxidase, lipooxygenase, cyclooxygenase (COX), xanthine oxidase (XO), uncoupled nitric oxide synthases (NOS), cytochrome P450, and mitochondrial respiration [12–14] (Figure 2). The process of increased O_2_^∙−^ generation, facilitated by XO enzyme, can be antagonized by a therapeutic approach with XO inhibitor, like allopurinol, to ameliorate cardiac conditions [15]. NADPH oxidase (Nox), commonly found on the cellular membrane, is stimulated during phagocytosis leading to increased ROS release [10]. In particular, the overexpression of Nox2 and Nox4 is linked to the remarkable oxidative stress observed during CVDs. A study done by Kuroda et al. showed that Nox4 knockout mice showed a low level of cardiac O_2_^−^ revealing that Nox4 is a potential source of superoxide in cardiac myocytes. Nox4 overexpression worsened the cardiac function and induced apoptosis and fibrosis in a mouse with response to pressure overload. Thus, Nox4 is a key contributor of oxidative stress in the mitochondrial redox systems leading to cardiac impairment during pressure overload. Therefore, the physiological role of Nox, translocating electrons throughout the membrane, can be deregulated in CVDs leading to cardiac dysfunction [16]. However, some pathways associated with ROS mediated CVDs are yet to be clarified. However, researchers are trying to reveal good, bad, and ugly roles of ROS in the physiological system. In contrast to the good face of ROS on signaling and immune response, at high concentrations, ROS can exhibit the deleterious effect on redox homeostasis leading to intracellular components damage as seen in neurodegenerative diseases, CVDs, and pulmonary disorders [5].

## 3. Oxidative Stress and Endothelial Dysfunction

Endothelial cells are lining the interior surface of blood and lymphatic vessels cells. Endothelial cells play an important role in homeostasis and immune and inflammatory reactions. EC regulates vascular tone by releasing various vasodilator factors such as nitric oxide (NO) endothelium derived hyperpolarizing factor, prostacyclin or vasoconstrictive factors such as thromboxane (TXA_2_), and endothelin-1 (ET-1). Endothelial dysfunction (ED) is a pathological state of the endothelium, which is a predictor of various CVDs, and is caused by imbalance between vasodilating and vasoconstricting substances [17]. ROS are considered as signaling molecules that contribute to ED in experimental and clinical atherosclerosis [3, 18]. NO is a potent vasodilator produced by the endothelium. Besides vasorelaxation, nitric oxide exerts various functions like antiplatelet, antithrombotic, and anti-inflammatory properties and permeability decreasing properties [19]. It is reported that ONOO^−^ which is formed by the reaction of superoxide and free radical NO can oxidize tetrahydrobiopterin. If formed in a small amount, ONOO^−^ exerts a similar physiological activity like NO. However, at a high concentration, it shows injurious activity by converting to harmful peroxynitrous acid and causing alteration of protein structure [20]. ED is associated with polymorphisms of various genes which include cytochrome P450, methylene tetrahydrofolate reductase, p22phox, angiotensin convertase enzyme, and glutathione-S-transferase [21]. Excess amount of ROS damages the endothelium, especially the terminal arteries leading to alteration of the intracellular reduction-oxidation homeostasis [22]. In a patient with diabetes mellitus, small vessel disease linked with mitochondrial disorders might also be due to oxidative stress. The result of diabetes mellitus in atherosclerosis is stimulated mainly by oxidative stress [23]. ROS may also activate mitogen activated protein kinase, which regulates the expression of monocyte chemoattractant protein 1 (MCP-1) and favors the chemotaxis of circulating monocyte to the site of atherosclerotic lesion. This demonstrates a potential link between arterial wall strain and atherosclerosis [24].

## 4. ROS in Ischemia-Reperfusion (I/R) Damage

Several researches support the fact that ROS are involved in ischemic occlusion leading to cardiac damage [39, 40]. Apart from carrying out the function of cellular O_2_ storage and supply by oxymyoglobin (Mb), it also acts as a potent preventive source against I/R injury [40]. During the outset of I/R injury, O_2_^∙−^ release has been observed in an isolated rat heart. Zhu and Zuo speculate that generation of O_2_^∙−^ is linked with Mb because of the lower myocardium oxygen tension. Results revealed that the rise of fluorescence in the ischemic heart was terminated by a SOD mimic, carbon monoxide (CO), or by Mb gene knockout. Likewise, O_2_^∙−^ was not formed in intracellular EC but rather from the myocytes, which are considered a potential source of Mb. This suggests that Mb is an important factor responsible for production of O_2_^∙−^ during ischemia [40]. An enzyme responsive to stress, named sirtuin-6 (SIRT6), displays cardiac protection from I/R injury as revealed in partial SIRT6 knockout mice as well as* in vitro* cultured cardiomyocyte. SIRT6 is a deacetylase and mono-ADP ribosyltransferase enzyme responsive to oxidative stress and can protect the cell against oxidative stress. This protective activity was achieved by initiating the expression of catalase and manganese SOD antioxidant-encoding gene resulting in reduced cellular oxidative stress [41]. Restoration of the coronary artery blood flow reverse to the ischemic myocardium can have a detrimental effect on the microvascular function, causing arrhythmias [42]. In the endothelium, the rise of ROS release and the opening of mitochondrial permeability transition (MPT) pore play an important role in the protection from I/R damage [43]. However, Kim and Lemasters showed that mitochondrial ROS, accompanied by normalization of pH, stimulate initiation of MPT pore followed by death of myocytes after reperfusion. However, Ca^2+^ overloading does not promote onset of MPT pore [44]. Research demonstrated that the reason behind the protective effect was the involvement of ROS and potent vasodilator NO in regulating downstream pathways by stimulating adenosine triphosphate sensitive potassium channel in mitochondria [45]. After open cardiac surgery, the I/R injury can influence postsurgical consequences because of the lipid peroxidation mediated by ROS [46]. In comparison to the heart obtained from juvenile rat, the cardiac dysfunction due to I/R was dramatically concealed in the heart obtained from congenital heart disease (CHD) model rats. Moreover, the ratio of n-3/n-6 PUFA was remarkably raised in I/R phase in CHD rats, whereas it was not observed in juvenile rats suggesting that the rise in n-3/n-6 ratio could result in the upregulation of cell defense system against oxidation via n-3 PUFA oxidation product 4-hydroxy-2-hexenal causing higher tolerance to I/R damage [46].

## 5. ROS and Atherosclerosis

Excess production of ROS plays an important role in inflammation, disturbed blood flow/abnormal shear stress, and arterial wall remodeling. ROS causes remodeling through proliferation of smooth muscle cell and increased inflammation [39]. Repeated continuous exposure to nonstreamline shear stress of arterial regions generates O_2_ induced by endothelial Nox resulting in adhesion of monocytes [47]. The upregulation of adhesion molecules including P-selectin, VCAM-1, and E-selectin causes further inflammation by adhesion of white blood cells. Development of inflammatory response increases ROS production by phagocytosis, which is important in the early stage of atherosclerosis [48, 49]. The Nox family of superoxide producing proteins is an important source of ROS in signal transduction. Nox are found to be expressed in phagocytic cells, EC, smooth muscle cells, and fibroblasts. Experiments conducted on arteries from human volunteers with coronary artery disease and animal experimental model with hypertension, diabetes, or atherosclerosis demonstrated that Nox1, Nox2, and Nox5 stimulate endothelial dysfunction, inflammation, and programmed cell death; however, isoform Nox4 protects the vascular system by increasing bioavailability of nitric oxide and stoppage of cell death pathways [50]. Some research presents the controversial role of Nox4 displaying either protective or a deleterious role of Nox4.

Nox4 are found abundantly in kidney, vascular cells, and osteoclasts [16]. Angiotensin II type 1 receptor activation and hypertension are linked to increased expression of Nox1 and Nox4 that could lead to vascular damage during chronic hypertension [51]. MCP-1 is essential for the formation of endothelial cell tumors (hemangioendotheliomas) which is redox sensitive. It was found that only the Nox4 isoform was present in endothelial cell tumors cells whereas knockdown of* Nox4* gene remarkably decreased the expression of MCP-1 as well as hemangioendothelioma formation. This was due to the fact that, in hemangioendothelioma cells, Nox4 delivers H_2_O_2_ to the nuclear compartment causing oxidative alteration of DNA [52]. Inflammation mediates all stages of atherosclerosis and ROS sources might include infiltrated monocytes/macrophages, dysfunctional EC, and smooth muscle cells that migrated from tunica media to tunica intima layers of the wall of an artery. ROS oxidized-LDL is available in the arterial wall and macrophages scavenge it resulting in the formation of foam cells. This is one of the important steps in the progression and development of atherosclerosis [53]. Also, the calcium-dependent zinc containing endopeptidase, matrix metalloproteinase, secreted from EC, foam cells, and vascular smooth cells, is activated during oxidative stress in part due to inflammation and nonlaminar shear stress, resulting in the ruptures of thrombosis [54].

## 6. Oxidative Stress and Mitochondria

Mitochondria play an important role in cellular signaling pathways, particularly in the modulation of calcium stores within the cell, generation of ROS, respiration, and biogenesis. So, changes in mitochondrial function lead to development of human diseases [55, 56]. Mitochondrial DNA (mtDNA) damage is linked to the atherosclerotic lesions in apolipoprotein E (apoE) knockout mice and also introduces atherogenesis in young apoE knockout mice [57]. Raised levels of mtDNA damage have been seen in the vascular tissue of CVD patients [58]. Mitochondrial dysfunction is due to decreased manganese SOD, increased damage of mtDNA, and increased atherosclerosis in apoE knockout mice [59]. There is excessive mitochondrial damage in atherosclerosis model. Oxidized-LDL stimulates mitochondrial complex I activity which depends on the induction of oxidative stress [60, 61]. Composed of 46 subunits, human mitochondrial complex I is the key enzyme responsible for oxidative phosphorylation. Dysfunction of the mitochondrial oxidative phosphorylation in a physiological system is responsible for occurrence of CVDs in humans, and mitochondrial diseases are linked to mitochondrial respiratory-chain pathologies and mutations of mitochondrial DNA. Studies reported that various stress induced in the cells causes structural and functional disturbance of mitochondria [62, 63]. Dysfunction of mitochondria provokes a signaling pathway for cell death resulting in organ failure and diseases. Mitochondria based pathological conditions including obesity, cancer, stroke, diabetes, neurodegenerative diseases, heart failure, and aging, however, are caused by intrusion of mitochondrial Ca^2+^, ATP, or ROS metabolism [61, 63]. Myocardial ischemia-reperfusion injury leads to mitochondrial Ca^2+^ overload and consequent generation of ROS and opening of the mitochondrial permeability transition pore [60–62], resulting in apoptosis. Compounds which can reduce mitochondrial Ca^2+^ overload, decrease mitochondrial ROS collection, and prevent mitochondrial energy generation are all potential sources of therapies for preventing disease. Mitochondria produce oxidative stress which plays an important role in mediating programmed cell death (apoptosis) and damage to mtDNA and leads to human aging, cancer, and CVDs. Oxidative damage of the mitochondrial membrane results in depolarization of membrane and uncoupled oxidative phosphorylation and altered cellular respiration. Altered mitochondrial respiratory chain can hinder the pivotal role of providing the energy to the cell as ATP, leading to various disease progression [60–64].

## 7. Methods for Detection of ROS in CVDs

Since ROS are highly unstable and very reactive, researchers always face the problem of precisely monitoring them in biological systems. One way to find out the possibility of ROS in CVDs subjects involves exploring experimental proof of oxidative reactions. Fluorescent probes and electron spin resonance probes tools for detection of ROS are limited in animal and human experiment due to technical problems [65]. The following list of direct methods can show at least in part indirect evidence of ROS effect in CVDs (Table 1).

### 7.1. Biomarkers of ROS

#### 7.1.1. Lipid Peroxidation Mediated by ROS

LDL collects in the blood vessels walls and lipid species undergo oxidation in the presence of several ROS [66]. It is reported that oxidative modification of LDL plays an important role in the atherosclerosis process [67]. Macrophages take up oxidized-LDL through scavenger receptor pathways resulting in cholesterol ester-rich foam cells and EC dysfunction, in part, by role of lectin-like oxidized-LDL receptor-1 [68, 69]. In atherosclerotic plaques, the availability of oxidized-LDL has been observed by using immunohistochemical staining for modified primary apolipoprotein B-100, the protein moiety in LDL [38]. Elevated levels of autoantibodies against oxidized-LDL or malondialdehyde-modified LDL particles are linked to atherosclerosis and coronary artery diseases (CADs) including acute coronary syndrome. In healthy people, circulating levels of oxidized-LDL can be identified by techniques such as ultrasound while in diseased subjects it is commonly seen in clinical case of CAD [70–72].

#### 7.1.2. MPO

High levels of the enzyme myeloperoxidase which produces hypochlorous acid (HOCl) are found in human atheroma and are an important predictor of CAD, as well as in patients with unstable angina and mitochondrial infarction (MI). Increased numbers of myeloperoxidase-expressing macrophages are found in eroded or ruptured plaques [73]. Also, MPO and hypochlorite-modified proteins are colocalized in atherosclerotic lesions. Also, in human study research, strong inverse relation occurs between MPO serum concentrations and brachial artery flow-mediated dilation, which is another clinical marker of atherosclerosis [73, 74]. So, lowering MPO levels could lower occurrence of CVDs. Human studies have demonstrated that humans with total or near-total deficiency of MPO have a lower chance of developing CADs [73]. Reduced expression of MPO by its gene promoter polymorphism showed reduced CAD manifestations but increased MPO expression by MPO gene promoter polymorphism demonstrated raised CAD [75]. Since the level of plasma MPO is sensitive to heparin dosing [76], neutrophil activation [77], and the procedure of collection [78], there is a need for development of an appropriate method for its sampling.

#### 7.1.3. Plasma F_2_-Isoprostanes

F_2_-isoprostanes are considered as the best biomarkers of oxidative stress status and lipid peroxidation in an* in vivo* model. F_2_-isoprostanes are found in an esterified form in normal biological tissues and are available in free form in biological fluids, demonstrating “physiological” levels of oxidative stress. The F_2_-isoprostanes might be produced from membrane phospholipids or circulating LDL [79, 80]. The generation of F_2_-isoprostanes can be via the action of several cell types like monocytes which are involved in atherosclerosis and the oxidized products have been restricted to a particular area within foam cells and atherosclerotic plaques individual specimens [81]. Various human studies have demonstrated a link between CAD and isoprostane levels [82]. Raised levels of isoprostanes in urine are an independent risk factor of CAD and are found to be increased in patients having unstable angina. Raised levels of isoprostanes are important markers of ischemic tissue injury, chronic heart failure, congestive heart failure, and cardiac remodeling [79, 82, 83]. Therefore, F_2_-isoprostanes could be used for the prediction of cardiovascular events.

## 8. Molecular Role of ROS in Muscle Contraction

During skeletal muscle contraction, ROS are generated which can affect muscle adaptation and function. Zuo et al. studied whether ROS are generated in the process of muscle contraction in isolated single skeletal muscle fibers (using* Xenopus laevis* muscle), as well as whether these ROS generated by contraction have an impact on fatigue development. To detect the ROS generation, myofibers were loaded with fluorescent probe (dihydrofluorescein-diacetate) which reacts with ROS to form fluorescein. Fluorescein signal was raised remarkably in both the first (42 ± 14%) and the third periods (39 ± 10%) of maximal tetanic contraction. However, with the treatment of reference antioxidant compound, ebselen, there was no rise of fluorescein during the second contractile period suggesting that ROS generation is high during contractile activity and antioxidant treatment can halt ROS production without any effect on myofiber contractility [84]. In spite of the various pathways of ROS generation, the study of key pathways of their production is still undergoing. In particular, ROS generation in response to exercise, hypoxia, and heat in the diaphragmatic skeletal muscle (a key muscle during respiration) is a topic of interest [85, 86]. During the state of heat stress, O_2_^∙−^ is generated by skeletal muscle which can be quantified by cytochrome *c* reduction as it is correlated with arachidonic acid metabolism. The blockage of enzyme phospholipase A_2_ using manoalide remarkably reduced O_2_^∙−^ release. However, neither the blockage of COX with nonselective COX inhibitor indomethacin nor the blockage of CYP P-450 contingent monooxygenase with SKF-525A reduces O_2_^∙−^ generation. In contrast, lipoxygenase blockage with common inhibitors cinnamyl-3,4-dihydroxy-*α*-cyanocinnamate and 5,8,11,14-eicosatetraynoic acid drastically halted the signal. Moreover, O_2_^∙−^ generation was notably reduced by diethylcarbamazine (5-LOX inhibitor) suggesting that metabolism of arachidonic acid involving LOX is a key mediator of generation of extracellular O_2_^∙−^ in skeletal muscle [86]. The role of ROS in myocardial I/R injury has been widely studied [87]. Vanden Hoek et al. proposed the generation of a high amount of ROS in case of ischemia before reperfusion by an* in vitro* experiment in isolated cardiomyocyte during simulated I/R. The fluorescent probes 2′,7′-dichlorofluorescein and dihydroethidium (DHE) were significantly oxidized during ischemia, revealing ROS production. After an hour of ischemia, reperfusion leads to further generation of OH^−^ and H_2_O_2_. In contrast, treatment of antioxidant compounds (1,10-phenanthroline and 2-mercaptopropionyl glycine) during ischemia injury halted oxidant production, raised the viability of cardiomyocytes, and opposed contraction following ischemia. The ROS production in response to residual O_2_ as in case of ischemia causes cellular injury observed in the reperfusion stage [88]. In a similar study on cardiomyocytes model of ischemia performed by Becker et al., an inhibitor of mitochondrial site III (myxothiazol) reduced oxidation. However, the inhibitor of mitochondrial site IV (cyanide) along with NOS inhibitor (nitro-L-arginine methyl ester), XO inhibitor (allopurinol), and Nox inhibitor (apocynin) showed no effect, suggesting that excessive O_2_^∙−^ production is observed in ischemia prior to reperfusion through ubisemiquinone area of the MET chain [89]. Another study suggests that sublethal H_2_O_2_ production in isolated cardiomyocytes during the period of simulated ischemia modulates cell death later in reperfusion step, mainly due to the burst of reperfusion oxidant [90].

## 9. Therapeutic Strategy Targeting ROS Sources in CVD

### 9.1. Antioxidant

Antioxidants are a prime choice to fight against ROS. Therefore, it is crucial to formulate the strategies to halt abnormal ROS production inside the human body as well as improve innate antioxidant protection capacity. A cross-sectional research performed by Lane et al. reported that dietary supplements of vitamins E, C, and A help to lower occurrence of peripheral arterial disease [91]. Regular consumption of diet rich in vegetables and fruits (as a source of antioxidant vitamins) lowers the prevalence of CVDs, and, globally, it is recommended for enough daily intake of vegetables and fruit [92, 93]. These antioxidants vitamins A, C, and E, CoQ10, lycopene, and quercetin have been studied to explore their therapeutic and/or preventative effects on ventricular remodeling, atherosclerosis, heart failure, myocardial infarction, and ischemia-reperfusion heart injury [94–97]. Various herbal plants such as* Nelumbo nucifera* [98],* Juglans regia* [99], and* Rumex nepalensis* [100] are a rich source of compounds exhibiting remarkable antioxidant activity along with cardioprotective activity. Among the antioxidants, the most commonly used vitamin C and vitamin E as a cardioprotective supplement are discussed in the following section also including the information about their failure to revert the CVDs in some study.

#### 9.1.1. Vitamin C

It is well known that vitamin C helps in regulation of blood pressure. A considerable study favors the notion that vitamin C restores high blood pressure related baroreflex dysfunction [101–103]. Some study revealed that oxidative stress decreases the baroreflex sensitivity leading to the constant hypertensive state. As shown in a hypertensive rat model by Botelho-Ono et al., treatment of vitamin C (150 mg/kg, IV) remarkably lowered heart rate, with improvement in baroreflex sensitivity compared to the untreated hypertensive group. Also, treatment of NADPH oxidase inhibitor apocynin (30 *μ*g/kg, intravenous) maintained baroreflex sensitivity revealing that ROS generated via NADPH oxidase pathway plays a key role in the modification of baroreflex sensitivity in hypertension, whereas treatment of antioxidants (vitamin C) restored this change [102]. Similarly, Nishi et al. [101] also reported that chronic administration of vitamin C at a dose of 150 mg/kg/day drastically lowers the mean arterial pressure (MAP) in a hypertensive rat's model as compared to vitamin C untreated rat. Furthermore, the study also revealed increased expression of angiotensin II type 1 (AT-1) receptor in vitamin C untreated hypertensive rat, with downregulation of AT-1 in the vitamin C-treated group. Likewise, in a human clinical trial, Bruno et al. showed that IV infusion of vitamin C (3 g, over 5 min) significantly reduces both sympathetic nerve activity and blood pressure in essential hypertension patients (*n* = 32) but not in normotensive patients (*n* = 20). This study highlights the notion that the decrease in heart rate and sympathetic nerve activity leading to reduced blood pressure after application with vitamin C was because of makeover of baroreflex function [103]. Endothelial dysfunction, a cause of CVDs, is corrected in a human study (*n* = 93) by treatment with vitamin C (2 g) alone or in combination with vitamin E (600 mg) as shown by Uzun et al. Results showed enhanced vasodilation following an endothelium dependent pathway in the radial artery of a subject with coronary artery disease receiving vitamin C and/or vitamin E [104]. Vitamin C promotes synthesis and deposition of collagen (type IV) in the basement membrane of EC, induces endothelial proliferation, scavenges radicals to prevent EC apoptosis, and increases endothelial NO production. However, there is variation in the beneficial effect of synthetic vitamin C and natural (food-derived) vitamin C on CVDs. One reason may be that, along with dietary vitamin C (from fruits and vegetables), we also consume other phytochemicals which may potentiate its availability to systemic circulation for action. This hypothesis is supported by a study done by Agarwal et al. on a human cohort. The results revealed that although vitamin C supplement did not help to decrease the progression of carotid artery intima-media thickness (IMT) in CVDs like atherosclerosis, dietary vitamin C did [105]. In contrast to the beneficial action of vitamin C to alleviate CVD symptoms, there is some controversy because of the results of the clinical trial which oppose this fact. For instance, a clinical trial done by Ward et al. showed that although monotherapy of vitamin C (for 6 weeks) decreases the systolic blood pressure in hypertensive subjects, combination therapy of vitamin C and grapes seed polyphenol increased it. Moreover, the endothelium independent and dependent vasorelaxation as well as oxidative stress marker were not significantly different from vitamin C/grape seed polyphenol therapy as compared to a hypertensive individual without therapy. This finding recommends that hypertensive individuals on vitamin C and polyphenol supplements therapy should take the necessary precaution [106].

#### 9.1.2. Vitamin E

Various studies have been carried out to examine the beneficial effect of vitamin E on CVDs. Serbinova et al. compared the effect of palm oil vitamin E with tocopherol alone and found that palm oil vitamin E was comparatively more successful in safeguarding the cardiac ischemia-reperfusion damage in the isolated heart [107]. A recent meta-analysis study done by Ashor et al. [108] to observe the potency of vitamins on atrial stiffness in adults disclosed that antioxidant vitamins possess a beneficial effect by reducing arterial stiffness. Moreover, the efficacy was dependent on the duration of treatment and dose supplemented. Those subjects having reduced levels of vitamin E in the blood attained an improved pharmacological effect from this intervention. In another double-blind, placebo-controlled study performed by Stephens et al. [109] on a coronary disease patient, it was found that 400 or 800 IU per day dose of vitamin E notably decreased the incidence of nonfatal myocardial infarction in study subjects.

### 9.2. Failure of Antioxidant Vitamin Therapy to Revert the CVDs

Although considerable research supports the fact that antioxidants possess therapeutic benefit to fight against disease progression, however, clinical trials are unsuccessful in showing the benefit [110]. Hasty et al. found that vitamin E supplementation for 12 weeks was not successful in alleviating the oxidative damage in western-type diet fed low density lipoprotein receptor knockout (LDLR−/−) mice model of obesity/hyperlipidemia. Although diet was enough to drastically increase the plasma lipid profile like free fatty acid, triglyceride, and total cholesterol (a marker of atherogenesis) in LDLR−/− obese mice with respect to lean mice, there was no beneficial effect observed after supplementation of vitamin E. Furthermore, there was no reduction in the urinary isoprostanes (a biomarker of oxidative stress) levels suggesting that vitamin E does not account for the cardioprotective effect [111]. A human clinical trial done in 730 volunteers (either sex, ≥65 years) for more than 20 years demonstrated that lower vitamin C supplementation was associated with high mortality rate by stroke in elderly people. However, there was no remarkable link between vitamin C diet status and CHD [112]. Similarly, another human clinical trial done in a larger population (6996 men and 2545 women) also showed that vitamin E had no beneficial effect on cardiovascular outcomes in patients who are more prone to cardiovascular events even after treatment for a long period of 4.5 years [113].

### 9.3. Pharmacological Agent

Various pharmacology agents such as statins and angiotensin-converting enzyme (ACE) inhibitors show pleiotropic effects to halt the oxidative stress. Particularly in the myocardium, oxidative stress and cell signaling proteins like Rac, Rho, and Ras are responsible for the cardiac hypertrophic response [114]. A recent* in vivo* investigation revealed that phagocyte-type Nox could be a potential source of ROS in the myocardium [115]. Nox-dependent ROS production seems to be linked with cardiac hypertrophy mediated by pressure overload [116] and angiotensin II infusion [117]. Even though the principal effect of statin therapy in CVDs is mainly vascular,* in vivo* studies recommend that there are also protective effects on the myocardium. Since Rac1 is essential for Nox function and cardiac hypertrophy resulted to some extent by oxidative stress, probably the statins could reduce cardiac hypertrophy by antioxidant pathway. Particularly, statins were successful in blocking angiotensin II-mediated oxidative injury in a rat model of cardiac hypertrophy [118]. Also, this activity of statins was seen in a clinical trial done in a cardiac hypertrophy subject that presented with hypercholesterolemia [119]. In a patient with heart failure, Nox mediated ROS generation raised in the left ventricular myocardium and associated with the rise in Rac1 GTPase activity, whereas statin therapy was able to reduce Rac1 activity in the heart [120].

#### 9.3.1. Statins

Statins category medicines are not a direct scavenger of ROS; however, they act in an indirect way by hindering the 3-hydroxy-3-methyl-glutaryl-coenzyme A (HMG CoA) reductase pathway involved in cholesterol synthesis. Metabolism with HMG CoA reductase leads to the production of intermediate pyrophosphates, considered as a crucial point for O_2_^∙−^ formation via Nox. The therapeutic effect of statins to lower the occurrence of CVDs is achieved by their capacity to promote endothelial nitric oxide synthase (eNOS) expression as well as antioxidant nature [118, 121–123]. Factors causing endothelial damage involve ROS such as oxidized-LDL and hypoxia can reduce the expression of eNOS whereas statins group medicine can reverse the eNOS downregulation, highlighting their ability to ameliorate the vessel NO bioavailability and atherosclerotic plaque stability [124–126]. Furthermore, statins also block tumor necrosis factor-*α* (TNF-*α*) mediated downregulation of eNOS [127]. Stimulation of angiotensin II hormone will further activate Rac1 and ADP-ribosylation factor 6 (ARF6), a controller of NADPH oxidase function. ARF6 is crucial for ROS production because, in the knocked down situation of this GTPase, angiotensin II cannot stimulate O_2_^∙−^ (superoxide anion) generation. Likewise, ARF6 also regulates NADPH oxidase 1 (Nox1) expression [128]. A study done by Copaja et al. showed that induction of cardiac myofibroblasts and fibroblasts apoptosis by simvastatin followed a cholesterol synthesis independent pathway but was dependent on Rho GTPases protein isoprenylation. On comparison, cardiac myofibroblasts were less sensitive to apoptosis induction than cardiac fibroblasts by simvastatin. Therefore, it is likely that simvastatin could circumvent harmful cardiac remodeling followed by some fibrotic restoration of the injured tissues [129]. Taken together, the abovementioned beneficial role of statins in prevention of coronary heart disease can promote the development of statin therapy against ROS mediated CVDs.

#### 9.3.2. ACE Inhibitors

ACE is an enzyme that metabolizes angiotensin I to angiotensin II. ACE inhibitors were designed to treat hypertension. During arterial wall remodeling, increased angiotensin II activity leads to thickening of the tunica media and narrowing of the vessel diameter, a key feature of atherosclerosis [130, 131]. In particular, increased angiotensin II level is associated with the proportional release of vascular O_2_^∙−^ [132]. In a rabbit model of hypercholesterolemia, it was observed that O_2_^∙−^ bioavailability was decreased in thoracic aorta due to the antioxidative activity of NO [133]. In contrast, NO concentration can decrease after reaction with O_2_^∙−^, thereby worsening the atheroma plague formation [134]. Although eNOS and neuronal NOS control normal metabolic functions, upregulation of inducible NOS leads to enhanced production of NO displaying deleterious inflammatory responses by forming peroxynitrite by NO and superoxide [135]. Circulating angiotensin II level can be enhanced by angiotensin II type I receptor blocker (ARB) leading to stimulation of angiotensin II type II receptors followed by vasodilation due to NO production [136, 137]. A clinical trial in hypertensive patients undergoing candesartan (a type of ARB) therapy displayed remarkable diminishing of carotid artery intima-media thickness, mainly because of augmented NO production as well as decreased oxidative stress [136]. Likewise, cotreatment of ACE and ARB inhibitor showed a synergistic inhibitory action against oxidative stress in a balloon-injured rat carotid artery [138].

## 10. Conclusions

The exact mechanism of CVD is complex and is not yet fully understood. ROS plays an important role in the progression and development of CVD. There is a link between ROS and the pathophysiology of CVD. We have developed a greater understanding of production of ROS, detection of ROS, and therapeutic strategy to prevent production of ROS and cardiovascular disease. However, more works to improve the detection and treatment of the ROS mediated dysfunction are necessary in the upcoming days.

## Figures and Tables

**Table 1:** Direct methods for detection of ROS in CVDs.

Methods	ROS detected	Applications/mechanism	Reference
Fluorescent protein-based redox probes	Cytoplasmic and mitochondrial H_2_O_2_	Used to detect redox status and ROS by introducing adenoviruses or plasmids inside cells. Afterwards, cells form chimeric proteins efficient to detect alteration in the redox status or ROS.	[25, 26]

Dihydroethidium (DHE) and mitochondrion-targeted probe mitoSOX	Cellular and mitochondrial O_2_^∙−^	Can detect mitochondrial O_2_^∙−^ by adding a triphenylphosphonium group for promoting its collection in the mitochondria. Similar to DHE, mitoSOX reacts with O_2_^∙−^ to give 2-hydroxy-mito-ethidium (2-OH-Mito-E^+^) so as to be identified and measured using HPLC.	[27–29]

Cyclic hydroxylamine spin probes	Total cellular and mitochondrial O_2_^∙−^	Allows measurement of O_2_^∙−^ in tissue, in *in vitro* cells, and *in vivo*.	[30–32]

Boronate-based fluorescent probes	H_2_O_2_ and ONOO^*∙*−^	As probes have a fluorophore which is secured by boronate, when subjected to H_2_O_2_, the boronate encounters a nucleophilic attack, followed by its displacement from the fluorophore, thus causing emission of light.	[33, 34]

Immunospin trapping	Free radical adduct formation in the mitochondria, cells, and tissue samples	5,5-Dimethyl-1-pyrroline-N-oxide reacts with protein radicals to form epitopes which can be particularly characterized immunologically.	[35, 36]

*In vivo* using X- and L-band ESR spectroscopy	Short-lived free radicals in whole living animals	Detection is done *in vivo* by infusion of cyclic hydroxylamines or nitrone spin traps, followed by *ex vivo* study of the tissue or blood using X-band (9 GHz) electron spin resonance spectroscopy.	[37, 38]
